# Computational Quantification of the Zwitterionic/Quinoid Ratio of Phenolate Dyes for Their Solvatochromic Prediction

**DOI:** 10.3390/molecules27249023

**Published:** 2022-12-17

**Authors:** Andrés Aracena, Moisés Domínguez

**Affiliations:** 1 Instituto de Ciencias Naturales, Facultad de Medicina Veterinaria y Agronomía, Universidad de Las Américas, Sede Santiago, Campus La Florida, Avenida Walker Martínez 1360, La Florida 8240000, Santiago, Chile; 2 Facultad de Química y Biología, Universidad de Santiago de Chile, Estación Central 9160000, Santiago, Chile

**Keywords:** solvatochromism, phenolate dyes, zwitterionic structure, quinoid, charge transfer

## Abstract

Solvatochromic dyes are utilized in various chemical and biological media as chemical sensors. Unfortunately, there is no simple way to predict the type of solvatochromism based on the structure of the dye alone, which restricts their design and synthesis. The most important family of solvatochromic sensors, pyridinium phenolate dyes, has the strongest solvatochromism. Using a natural population analysis (NPA) of the natural bond orbitals (NBO) of the phenolate group in the frontier molecular orbitals, it is possible to calculate the relative polarity of the ground state and excited state and, thus to develop a model that can predict the three types of solvatochromism observed for this family: negative, positive, and inverted. This methodology has been applied to thirteen representative examples from the literature. Our results demonstrate that the difference in the electron density of the phenolate moiety in the frontier molecular orbitals is a simple and inexpensive theoretical indicator for calculating the relative polarity of the ground and excited states of a representative library of pyridinium phenolate sensors, and thus predicting their solvatochromism. Comparing the results with the bond length alternation (BLA) and bond order alternation (BOA) indices showed that the NPA/NBO method is a better way to predict solvatochromic behavior.

## 1. Introduction

Solvatochromic dyes have been used for years to gather information about chemical and biological systems [1,2,3,4,5,6,7,8] because the intramolecular charge transfer in this class of molecules shifts accompanying medium polarity changes. Depending on the direction of this shift, three types of solvatochromic behavior have been recognized in the literature: negative, positive, and inverted solvatochromism [9,10]. When the medium polarity increases, negative solvatochromic dyes progressively displace their absorption band to shorter wavelengths (hypsochromic shifts). In contrast, if a progressive change to a longer wavelength of the absorption band of the dye is observed (bathochromic shifts), the compound is classified as a positive solvatochromic dye. Finally, inverted solvatochromic dyes shift from positive to negative solvatochromic behavior at a specific polarity value, called the inversion point.

**Figure 1:**
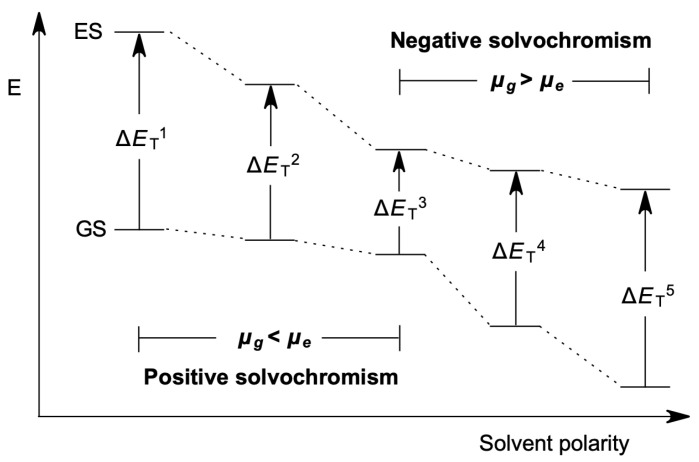
Schematic representation of the influence of the solvent polarity on electronic transition energy in dipolar solvatochromic dyes [11,12].

The pair of phenolate/electron-poor heterocycles are the most studied motif due to the significant solvatochromic ranges they usually exhibit [10]. The chemical structure of these dyes can be represented by the joint of a donor and an acceptor fragment in the general formula X–Y. Charge transfer in this class of dyes goes from one side of the molecule to the other, causing a substantial difference in the dipolar moment between the ground (μ_g_) and the excited state (μ_e_). Indeed, solvatochromism has traditionally been explained based on the relative stabilization of these two electronic states (Figure 1) [11,12]. As shown in Figure 1, negative solvatochromic dyes exhibit a more polar ground state than their corresponding excited state (μ_g_ > μ_e_). Therefore, when the solvent polarity increases, the energy gap between these two states also increases due to ground state stabilization (Δ*E*_T_^3^ < Δ*E*_T_^4^ < Δ*E*_T_^5^ in Figure 1). In contrast, if the excited state is more polar than the ground state of the dye, a systematic decrease in the energy gap will be observed because of the higher excited state stabilization, causing positive solvatochromism (Δ*E*_T_^1^ > Δ*E*_T_^2^ > Δ*E*_T_^3^ in Figure 1). Although the reasons for inverted solvatochromism are still unknown, this behavior has also been interpreted under this traditional model, postulating an inversion of the relative polarity between the ground and the excited state, passing from μ_g_ < μ_e_ in the positive solvatochromic part of the curve to a μ_g_ > μ_e_ in the negative part [13,14,15,16,17].

The schematic model shown in Figure 1 [11,12] serves for a posteriori interpretation of the experimental data, but cannot be easily applied to predict the spectral behavior of novel dyes because, in most cases, it is not easy to determine whether the ground or excited state is the most polar state.

**Figure 2:**
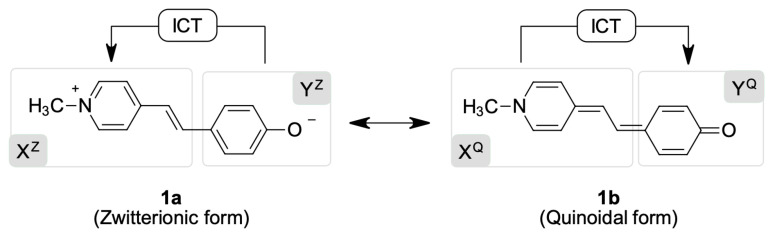
The two mesomeric limits of Brooker’s merocyanine **1** [21], an inverted solvatochromic dye. Superscripts Z and Q refer to the zwitterionic and quinoidal forms, respectively. ICT = internal charge transfer.

Solvatochromic dyes (of general formulae X–Y) are highly conjugated molecules and can display a continuum variation of their ground state structure; for example, the ground state of Brooker’s merocyanine **1** (Figure 2) can be represented by the zwitterionic limit **1a** (X^Z^–Y^Z^), the low polar quinoid-like structure **1b** (X^Q^–Y^Q^), or a polymethine-like structure between them. In fact, according to the traditional model (Figure 1), an inverted solvatochromic dye such as **1** should exhibit a less polar ground state (similar to **1b**) than its corresponding excited state (similar to **1a**) in low-polarity media where positive solvatochromism dominates. The charge-transfer band, in this case, will show an electronic density flow from the 1-methyl-1,4-dihydropyridine moiety X^Q^ to the 2,5-cyclohexadien-1-one moiety Y^Q^. In contrast, in the negative solvatochromic part of the experimental curve, the ground state should exhibit a more polar structure (similar to **1a**) than its corresponding excited state (similar to **1b**), with a charge transfer flow from the phenolate Y^Z^ to the *N*-methylpyridinium X^Z^. The variations in the ground state of solvatochromic dyes due to medium polarity changes have received computational [13,14,15,16,17] and experimental support [18,19,20].

Quantum-mechanics calculations have been employed to relate the structure and solvatochromic behavior of phenolate dyes. We have reported relationships between the three types of solvatochromism exhibited by phenolate-based dyes with their chemical hardness [22], electrophilicity [23,24], and Fukui functions [25]. None of these approaches has been able to predict the solvatochromic behavior of a large library of dyes without outlier cases, and none of these models have a chemical relationship with the traditional model typically employed to rationalize the solvatochromic tendency of a dye. As far as we are concerned, there have been no successful attempts to apply the model depicted in Figure 1 to the computational prediction of the solvatochromic tendencies of a large collection of dyes. BLA methodology has been applied with outstanding qualitative reproduction of the inverted solvatochromism of Brooker’s merocyanine **1** [26,27]. Moreover, whereas the BLA index is considered merely a measure of the geometric structure of molecules in conjugated systems, the BOA incorporates information about the electronic structure of pi-conjugated molecules [28]. When positive BLA values or negative BOA values are obtained from a molecule’s computation, the molecule is said to be in a neutral or quinoid ground state. On the other hand, if the BLA value is found to be negative and the BOA value is found to be positive, this indicates that the ground state of the molecule is either charge-separated or zwitterionic. However, solvatochromic dyes have not been evaluated using this methodology. Natural population analysis (NPA) of the natural bond orbitals (NBO) is still another method for computationally evaluating the model depicted in Figure 1, as it permits the calculation of the relative polarity of the ground state of a phenolate solvatochromic dye as the ratio of the two mesomeric limits that represent its structure.

This study applied the BLA and BOA indices and the NPA/NBO approach to a collection of thirteen representative solvatochromic dyes. We demonstrate that the BLA and BOA are extremely sensitive to their definition and application to the molecular structure of the dye. In contrast, the application of natural population analysis (NPA) to natural bond orbitals (NBO) is a superior index, allowing for the computer reproduction of the conventional model depicted in Figure 1.

## 2. Results and Discussions

### 2.1. The Library Employed in This Work

**Figure 3:**
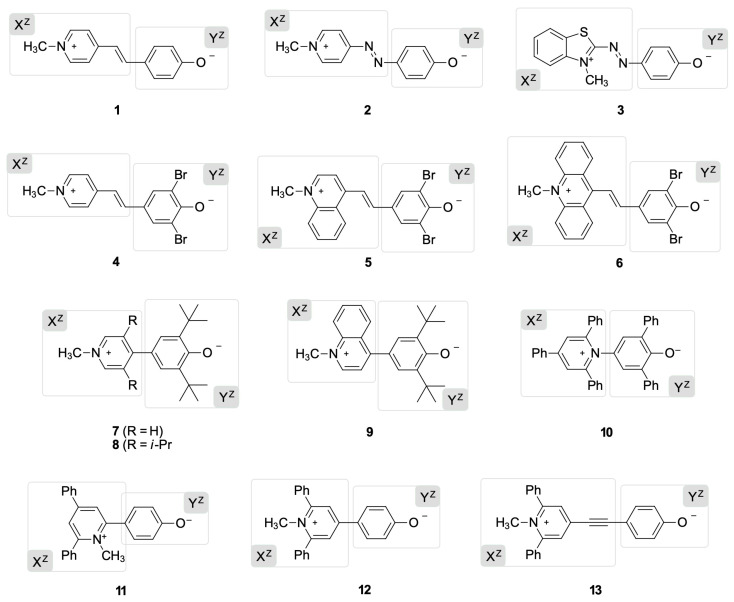
Molecular structures of the solvatochromic phenolate dyes X–Y studied in this work. All molecules are represented with their canonical zwitterionic formulae (Z), indicating how they were split into the X^Z^ and Y^Z^.

We applied our theoretical protocols to the thirteen phenolate solvatochromic dyes shown in Figure 3. The library includes negative, positive, and inverted solvatochromic dyes.

Brooker’s merocyanine **1** was the first inverted solvatochromic dye reported in the literature with a solvatochromic inversion in chloroform solution [21]. Dye **2** is the azo version of **1** and shows a positive non-linear solvatochromic behavior without an explicit solvatochromic inversion [29]. Benzothiazolium **3** is a positive solvatochromic dye, showing the same azo bridge as dye **2**, but a more electron-rich and annulated acceptor moiety [29]. The solvatochromic behavior of **4**–**6** varies with the increase in the annulation of the acceptor moiety X, passing from a truly negative solvatochromic behavior in **4** to a behavior represented by a negative non-linear solvatochromic behavior for **5** [30]. Finally, dye **6** with the more annulated acceptor part of the series exhibits a clear inverted solvatochromism with an inversion point in 2-butanol solution [30].

The solvatochromism of **7**–**9** varies with the degree of coplanarity between the phenolate and pyridinium groups [31], from a genuinely negative solvatochromism for dye **7**, which presents the most hindered R substituents (R = *i*-Pr), to a non-linear negative solvatochromic behavior in the less steric hindered dye **8** (R = H). The analog **9** reported by Barzoukas et al. [32] is a more annulated version of dye **8** and displays inverted solvatochromism, with an inversion point in dimethyl sulfoxide solution. Dye **10**, known as Reichardt’s betaine, is a negative solvatochromic dye widely used as a polarity sensor, whose behavior has been extensively reviewed [9,10]. Finally, dyes **11**–**13** are three solvatochromic dyes that show negative non-linear solvatochromic behavior [33,34].

A few years ago, we called attention to this very scarce class of compounds, such as **2**, **8**, and **11**–**13**, which do not display linear solvatochromic or inverted behavior, proposing a new classification of them as borderline solvatochromic dyes [34]. Therefore, we included them in our list as a challenging test for our protocol, trying to predict the spectral tendency of a group of dyes with an intermediate solvatochromic behavior. However, to maintain the traditional classification of three types of solvatochromism (negative, positive, and inverted), we decided to leave these dyes as positive or negative in Table 1 because they do not show a clear point of inversion.

### 2.2. The BLA and BOA Indices for Solvatochromic Tendency Predictions

**Figure 4:**
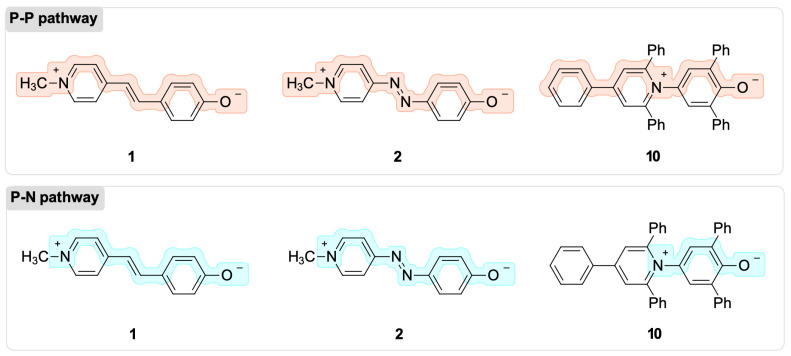
The two molecular pathways employed for the computation of the BLA and BOA indices for dyes **1**, **2**, and **10**, which are representative examples of inverted, positive, and negative solvatochromism, respectively.

First, we determined the BLA and BOA indices for a set of dyes typical of each of the three possible solvatochromic tendencies. For example, dyes **1**, **2**, and **10** correspond to inverted, positive, and negative solvatochromism. Unfortunately, there is no universal definition of how many atoms in the π-systems of a conjugated system need to be taken into account in order to calculate the BLA and BOA indices. As a result, we determined the two molecular pathways necessary for the computation of BLA and BOA, which are depicted in Figure 4. Both routes incorporate the nitrogen atom of the pyridinium ring and the oxygen atom of the phenolate; however, the number of atoms of the acceptor moiety that are factored into the computation of the indices are different for the two routes. Based on the fact that the charge-transfer band of solvatochromic dyes results from an electronic density flow change from the donor to the acceptor group of the molecules, these two mechanisms are possible. Depending on the nature of the ground-state structure of the dye, these two groups are represented by the X^Q^/Y^Q^ or X^Z^/Y^Z^ fragment pairs, but always with an oxygen and nitrogen-containing substructure (Figure 2). The P–P pathway is the one that connects the phenolate moiety to the pyridinium moiety, while the P–N pathway only connects the phenolate moiety to the positively charged nitrogen.

The application of the BLA and BOA indices to phenolate-based solvatochromic dyes could determine whether the dyes’ ground state is zwitterionic or quinoidal. According to the definitions of these indices, zwitterionic ground states have negative BLA values and positive BOA values, whereas quinoidal ground states have positive BLA values and negative BOA values. Table 1 shows the BLA and BOA values that were calculated for Brooker’s merocyanine **1**, azo-Brooker’s merocyanine **2**, and Reichardt’s betaine **10** in four medium polarities, from gas to water.

The results in Table 1 show positive BLA values and negative BOA values for dye **1** along the solvent polarity range studied when the P–P pathway is considered for the calculation.

The obtained BLA and BOA values indicate a predominance of the quinoidal character in the ground-state structure of the dye (X^Q^–Y^Q^). Thus, the BLA and BOA indices for the P–P pathway wrongly predicted the solvatochromic inverted dye **1** as positive. Nevertheless, when the P–N pathway is employed, the change from a quinoidal (X^Q^–Y^Q^) to a zwitterionic (X^Z^–Y^Z^) ground-state structure is observed as an inversion in the sign of the calculated values in the passage from the gas phase to chloroform (CH_3_Cl). When the P–N pathway is employed, the BLA and BOA indices correctly predict the experimental solvatochromic tendency observed for dye **1** as an inverted solvatochromic dye.

Only positive BLA values were observed for dye **2** in the passage from the gas phase to water via the P–P pathway (Table 1), indicating that a quinoidal ground-state structure (X^Q^–Y^Q^) predominates over the entire solvent polarity window. The same conclusion was obtained from the calculated BOA values, which showed a negative sign in all of the media. Therefore, both BLA and BOA accurately anticipated that dye **2** was a positive solvatochromic compound due to its quinoidal ground-state structure. The opposite conclusion was reached, however, when the P–N route was employed. Both the BLA and BOA indices incorrectly predicted a charge-separated or zwitterionic ground-state structure (X^Z^–Y^Z^) for dye **2**, which is typical for dyes with negative solvatochromism.

In the transition from the gas phase to the continuum, the application of the P–P pathway for dye **10** revealed negative BLA values and positive BOA values, indicating a zwitterionic ground-state structure and a negative solvatochromic behavior. Nevertheless, if we use the P–N route, the BLA and BOA values indicate a neutral or quinoid structure in the transition from the gas phase to water, incorrectly predicting the iconic negative solvatochromic dye **10** to be a positive solvatochromic dye.

In conclusion, the results for dyes **1**, **2**, and **10** (Table 1) demonstrate that the BLA and BOA indices are highly dependent on the chemical pathway considered during their calculation. For example, the BLA and BOA indices in the P–P pathway accurately predicted the solvatochromic tendency of positive dye **2** and negative dye **10**, but incorrectly anticipated that inverted dye 1 is a positive solvatochromic dye. Alternately, if the P–N route is taken into account, the same indices accurately predict the inverted solvatochromism of **1**. To overcome these ambiguities, we decided to investigate the natural population analysis (NPA) of the natural bond orbitals (NBO) as an alternative computational method for solvatochromic predictions of the phenolate-based dyes depicted in Figure 3.

### 2.3. Prediction of the Solvatochromic Tendencies by the Natural Population Analysis (NPA) of the Natural Bond Orbitals (NBO)

Equation (1) [35] can be employed to quantify the characterization of the electronic transition of a dye as a partial charge-transfer (CT) in terms of the percentage of participation of the molecular subunits X and Y of a molecule with the general formula X–Y.

CT (%) = 100 × (P_Y_^g^ − P_Y_^e^) (1)

We defined Y as the electron-donor fragment during the electronic transition, and P_Y_^g^ and P_Y_^e^ as the electronic densities of fragment Y in the ground state and the excited state, respectively. Equation (1) can be rewritten using the atomic orbital contributions to the molecular orbitals involved in the electronic transition [36]. In particular cases such as the dyes studied here (see Supplementary Materials), exhibiting the HOMO–LUMO transition as the primary transition in the pass from S_0_ to S_1_, the CT character in the percentage of the fragment Y can be defined as:

CT (%) = %Y_HOMO_ − %Y_LUMO_ (2)

The compositions in the percentage of molecular orbitals between fragments can be easily obtained using the AOmix software, which uses the information from the previously computed NPA calculations. This information for the Y fragment of a dye can be employed in Equation (2) to obtain the CT values, and subsequently, a solvatochromic tendency prediction for a dye after employing various solvent permittivity values.

The ICT of all dyes in our library involved HOMO → LUMO as the primary electronic transition responsible for the solvatochromic band observed experimentally. This nature of the electronic transition was validated using a DFT-level spectrum computation. Representative examples of the calculation of natural transition orbitals (NTOs) for dyes **1** (inverted), **2** (positive), and **10** (negative) in the gas-phase can be found in the Supplementary Materials. Therefore, the zwitterionic (X^Z^–Y^Z^) or quinoidal (X^Q^–Y^Q^) character of the ground state of the phenolate dyes shown in Figure 3 (shown as zwitterionic canonical formulae, their quinoidal canonical formulae can be seen in the Supplementary Materials) can be quantified by computing the participation of the atomic orbitals of fragment Y in the HOMO and the LUMO of the whole molecules according to Equation (2). Thus, if fragment Y exhibits higher participation in the ground state (%Y_HOMO_ > %Y_LUMO_) across the entire polarity range, the resulting dye will display negative solvatochromism, with electronic flow from the phenolate moiety to the positively charged heterocycle as a consequence of the predominant zwitterionic character of this state, a situation represented by the X^Z^–Y^Z^ formula. In contrast, if fragment Y shows higher participation in the LUMO of the dye throughout the entire solvent polarity range, positive solvatochromism will be observed because of a quinoidal structure predomination in the ground state, a situation represented by the X^Q^–Y^Q^ formula. In this last case, the electronic charge will flow from the neutral heterocyclic subunit (X^Q^) to the 2,5-cyclohexadien-1-one moiety (Y^Q^). Finally, inverted solvatochromic dyes will show a pass from CT negative values to CT positive values at a specific medium polarity.

We applied this protocol to compounds **1**–**13** in the gas phase and three solvents of increasing polarity: chloroform, dimethyl sulfoxide, and water. As seen in Table 2, varying the polarity of the medium led to a progressive variation in CT values.

The variation in the calculated CT values with the solvent polarity predicted the correct spectral tendency for inverted solvatochromic dyes **1** and **6**, negative solvatochromic dyes **4**–**5**, **7**–**8**, and **10**–**13** as well as positive solvatochromic dyes **2** and **3**.

Reichard’s betaine **10** is a paradigmatic example of a pyridinium phenolate dye that shows negative solvatochromism [9,10]. The increase in the positive CT value obtained for dye **10** when the polarity of the medium increased (Table 2) shows an unequivocal negative solvatochromic behavior because of a highly zwitterionic ground state (X^Z^–Y^Z^). Interestingly, the CT values obtained for dye **10** were the largest of the set studied, classifying **10** as the dye with the most zwitterionic ground state in the library. Dyes **11**–**13** exhibited negative solvatochromic behavior [33,34], and their solvatochromic tendency was well-predicted by our theoretical model, with CT values accompanying the increase in the polarity of the medium. Finally, the CT values obtained for dyes **7** and **8** assigned them as negative solvatochromic compounds, again, in agreement with the spectral behavior they showed experimentally [31,32].

The pass from negative to inverted solvatochromism by increasing the annulation of the acceptor moiety in pyridinium-phenolate dyes has been reported [30], and recently, we have demonstrated that this arises from an increase in the sensitivity of the dye to solvent polarizability [38]. In series **4**–**6**, the solvatochromic behavior was modified by the increment in the annulation of the acceptor moiety, passing from negative to inverted solvatochromism, all being well predicted by our protocol.

**Figure 5:**
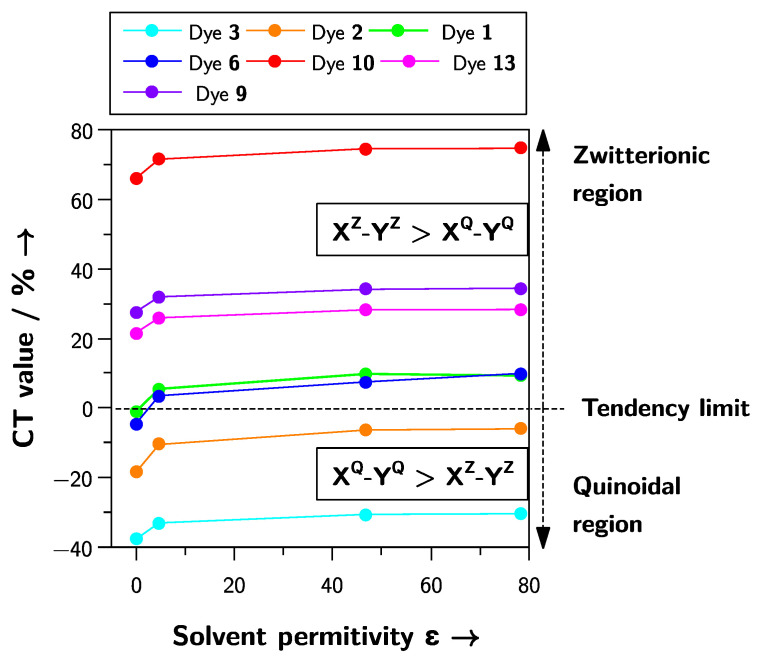
Difference in the percentage participation of the phenolate moiety between the zwitterionic and quinoidal mesomeric structures of dyes **1**–**13**.

In the present work, we proposed the percentage of participation of phenolates in HOMO and LUMO as a measure of the zwitterionic (X^Z^–Y^Z^) or quinoidal (X^Q^–Y^Q^) character of the ground-state structure of the dye. As shown in Figure 5, a variation in this percentage [(X^Q^–Y^Q^)-(X^Z^–Y^Z^)] with the medium polarity increase reveals the solvatochromic tendency of a dye, but also provides information about how zwitterionic or quinoidal the dyes in the set are. In Figure 5, the solvatochromic inversion of dyes **1** and **6** occurred in the pass from the gas phase to chloroform solution, which is in agreement with the experimental observation for **1**, which exhibited the solvatochromic inversion in chloroform solution. However, the solvatochromic inversion for dye **6** occurred in 2-butanol solution, a more polar medium. It is important to note that the solvation models available for the type of calculations our protocol requires are implicit solvation models that neglect the specific solute–solvent interactions (i.e., hydrogen bonds). These solute–solvent interactions indeed modulate the final solvatochromic response of a dye. Our protocol aims to classify dyes by predicting their experimental solvatochromism, and in the case of solvatochromically inverted dyes, predicting this behavior as a pass of the CT values from the quinoidal region (low part of the plot in Figure 5) to the zwitterionic region (up part of the plot in Figure 5), regardless of the accuracy of the polarity where this inversion takes place.

The interplanar angle between the X and Y moieties controls the electronic coupling between these fragments, modifying the energy required for the charge transfer from one group to the other [31,39]. A high interplanar angle between the X and Y fragments will increase the charge separation, increasing the zwitterionic character of the ground state, ultimately showing the tendency of the dye to display negative solvatochromism. In this context, dye **9** is a pathological case because it exhibited a well-defined inverted solvatochromism [32]. Dye **9**, whose optimized structures exhibited a high dihedral angle in the gas phase (ca. 25° in the gas phase) and water (ca. 29°), was wrongly predicted as a negative solvatochromic dye in our protocol. Regardless of whether the solvatochromism of dye is an exceptional case, there is a bias in our protocol regarding the solvatochromic prediction of dyes presenting high interplanar angles between the X and Y moieties, with all the molecules X–Y predicted as negative solvatochromic dyes.

**Figure 6:**
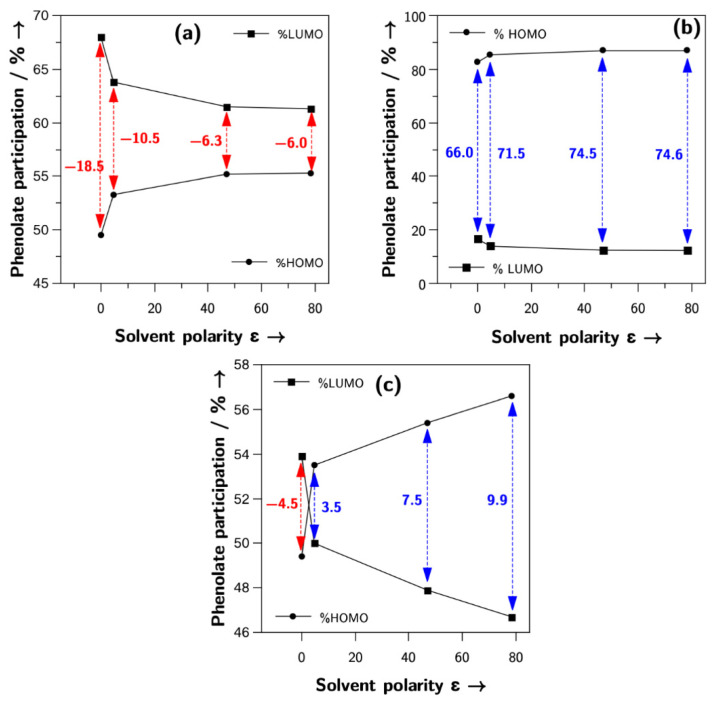
Percentage contribution of the phenolate group in the HOMO and LUMO orbitals for dye **2** (**a**), positive, dye **10** (**b**), negative, and dye **6** (**c**), inverted. CT values are highlighted in red for positive solvatochromism and blue for negative solvatochromism.

The scheme of the influence of solvent polarity on the electronic transition energy in the dipolar solvatochromic dyes shown in Figure 1 is a model commonly employed to explain the solvatochromic tendency of the dye. Although the three types of solvatochromism exhibited by phenolate-based dyes have been computationally correlated with their chemical hardness [22], electrophilicity [23,24], and Fukui functions [25], they depart from the traditional model shown in Figure 1. The protocol we propose in the present work, in combination with high predictive power, is a computational adaptation of this model. The CT values are a very straightforward way to calculate the zwitterionic degree of the ground state of a dye, providing the possibility to extend the ideas the literature has been using for a long time to understand solvatochromism by a simple computational protocol. In Figure 6, the application of our model to three dyes exhibiting positive (Figure 6a), negative (Figure 6b), and inverted solvatochromism (Figure 6c) is illustrated.

The ground-state structure of all dyes **1**–**13** was a mixture of X^Z^–Y^Z^ and X^Q^–Y^Q^, with no pure zwitterionic or quinoidal character; therefore, the variation in the Y group contribution to the HOMO and LUMO followed a very similar pattern to the one shown in the traditional model of Figure 1. The positive solvatochromic dye **2** exhibited a highly quinoidal ground-state structure X^Q^–Y^Q^ in the gas-phase, which slightly decreased with the medium polarity. In contrast, the negative solvatochromic dye **10** displayed a highly zwitterionic ground-state X^Z^–Y^Z^ in the gas phase, which became even more zwitterionic with medium polarity. Finally, the inverted solvatochromic dye **6** started as a mildly quinoidal structure X^Q^–Y^Q^ and became moderately zwitterionic X^Z^–Y^Z^ with the increase in the solvent polarity.

## 3. Materials and Methods

Molecular geometries of phenolate dyes **1**–**13** (Figure 3) were optimized at the density-functional theory (DFT) level with the hybrid functional B3LYP [40] and the 6-31G(d) basis set. The solvent effect was mimicked with the polarizable continuum model (PCM) [41] for the chloroform (ε = 4.71), dimethyl sulfoxide (ε = 46.83), and water solution (ε = 78.36). The BLA, BOA, and NTO calculations for representative dyes **1**, **2**, and **10** were obtained using Multiwfn software version 3.8 [42].

After optimizing the structures in the gas phase or continuum medium, the molecules X–Y were split into X and Y fragments according to Figure 3. Natural population analysis (NPA) of the natural bond orbitals of X–Y dyes, and their fragments X and Y were performed with the B3LYP/6-31G(d) method. Geometry optimization and population analysis calculations were carried out with the Gaussian09 package [43]. Finally, the molecular orbital compositions of the dyes in terms of their constituent chemical fragments X and Y were calculated with AOMIX software version 6.6 [44].

## 4. Conclusions

In this work, we report on a novel protocol for the computational prediction of the solvatochromic tendency of pyridinium-phenolates, the most important family of solvatochromic dyes. We applied this protocol to a library of thirteen representative examples from the literature. Calculating the electronic density contribution of the phenolate group (Y) to the HOMO and LUMO of the dye makes it possible to estimate whether the ground-state structure of the dye is quinoidal or zwitterionic. Furthermore, the variation in CT values with the increase in the medium polarity serves as a computational prediction of the experimental solvatochromism displayed by pyridinium-phenolates. CT values varied significantly between dyes **1** and **13**, especially when compared to Reichardt’s dye **10**, which has a broader solvatochromic range reported. Despite this observation, a relationship between the dye’s CT value and the experimental HOMO–LUMO gap could not be established due to the absence of explicit solute–solvent interaction in our calculations. The continuum solvent model used here allows for the simulation of some solvent-caused dye polarization, and even if these are the only options affordable at the quantum level, more is needed to encompass all of the solvent’s polarization effects [45]. Moreover, we have recently shown that dyes with different forms of solvatochromism are sensitive to different solvent properties [38].

Another advantage of this method is the possibility of quantifying the degree of zwitterionic/quinoidal character of a dye, a parameter that can be employed in designing novel dyes. Unfortunately, there is no easy way to anticipate the type of solvatochromism based solely on the structure of the dye, and although the vast literature on solvatochromism is still a black box, where, for instance, the kind of solvatochromism is most of the time subject to a posteriori verification and rationalization, limiting the design of novel and better sensors. Our results take a step toward solving this problem by showing that the difference in the electron density of the phenolate moiety in the frontier molecular orbitals provides a simple and inexpensive theoretical indicator for solvatochromic predictions.

The results presented here are based on the UV–Vis absorption process of a library of solvatochromic sensors, an electronic process where the molecular geometry of the dyes remains the same in the ground, and the excited state in each medium studied. Nevertheless, the idea of employing the natural population analysis (NPA) of the natural bond orbitals (NBO) as a method to quantify the charge-separated degree of a state could be extended to excited state processes, if the molecular geometry of the dye is optimized in the excited state prior to applying the NPA/NPO analysis. Therefore, the model presented here could be employed to rationalize solvent-dependent shifts in the emission spectra of organic molecules [46,47,48,49,50,51]. Furthermore, the model presented here could be applied to determine the dominance of the electron transfer process in excited states, an essential aspect for developing turn-off sensors based on emissive ligands that discriminate between analytes [50,51].

## Figures and Tables

**Table 1:** BLA (Å) and BOA index values for solvatochromic dyes **1** (inverted), **2** (positive), and **10** (negative) computed with the two pathways, P–P and P–N, as shown in Figure 4.

***BLA Index***						
**Dye**	**Solvatochromism**	**Pathway**	**Gas-Phase**	**CHCl_3_**	**Me_2_SO**	**H_2_O**
**1**	Inverted	P–P	0.064	0.044	0.032	0.034
		P–N	0.083	−0.062	−0.049	−0.051
**2**	Positive	P–P	0.074	0.058	0.049	0.049
		P–N	−0.098	−0.082	−0.072	−0.072
**10**	Negative	P–P	−0.027	−0.017	−0.013	−0.013
		P–N	0.088	0.069	0.062	0.062
***BOA Index***						
**Dye**	**Solvatochromism**	**Pathway**	**Gas-Phase**	**CHCl_3_**	**Me_2_SO**	**H_2_O**
**1**	Inverted	P–P	−0.302	−0.266	−0.240	−0.244
		P–N	−0.408	0.369	0.341	0.345
**2**	Positive	P–P	−0.317	−0.290	−0.273	−0.272
		P–N	0.421	0.391	0.373	0.372
**10**	Negative	P–P	0.051	0.033	0.025	0.025
		P–N	−0.144	−0.112	−0.097	−0.096

**Table 2:** Percentile difference between the electronic density participation of fragments X and Y in the HOMO and LUMO (CT) of dyes with the general formula X–Y.

Dye	Solvatochromism	Gas-Phase	CHCl_3_	Me_2_SO	H_2_O
**1**	Inverted	−1.0	5.5	9.8	9.3
**2**	Positive	−18.5	−10.5	−6.3	−6.0
**3**	Positive	−37.7	−33.0	−30.6	−30.4
**4**	Negative	4.9	13.2	17.4	17.7
**5**	Negative ^1^	6.3	15.5	21.0	21.3
**6**	Inverted	−4.5	3.5	7.5	9.9
**7**	Negative ^1^	38.6	44.2	47.3	47.5
**8**	Negative	41.7	48.2	52.5	52.8
**9**	Inverted	27.6	31.9	34.1	34.4
**10**	Negative	66.0	71.5	74.5	74.6
**11**	Negative ^1^	51.4	57.0	59.7	59.8
**12**	Negative ^1^	33.4	37.1	38.7	38.8
**13**	Negative ^1^	21.6	25.9	28.2	28.3

^1^ The experimental solvatochromism of this dye shows a parabolic curve but without a clear inversion point. Here, we classified the dye as a negative solvatochromic compound, but this kind of spectral behavior has been proposed as borderline solvatochromism [34,37].

## Data Availability

Not applicable.

## Supplementary Materials

The following supporting information can be downloaded at: https://www.mdpi.com/article/10.3390/molecules27249023/s1, a protocol of the %CT calculation and the fragment percentage of electron densities in the HOMO and LUMO for all dyes as well as the structure of the dyes used in this work when they are in their quinoidal form.
