# Supplementary material for: Computational Quantification of the Zwitterionic/Quinoid Ratio of Phenolate Dyes for Their Solvatochromic Prediction
Source: Molecules. 2022 Dec 17;27(24):9023. doi: 10.3390/molecules27249023 (PMC9782446; doi:10.3390/molecules27249023)
Supplement: Supplementary file 1 [file molecules-27-09023-s001.zip › molecules-2054068-supplementary.pdf]

Computational quantification of the zwitterionic/quinoid ratio of phenolate dyes for their solvatochromic prediction

Andrés Aracena,*[,a] and Moisés Domínguez*[,b]

[a] Instituto de Ciencias Naturales, Facultad de Medicina Veterinaria y Agronomía, Universidad de Las Américas, Sede Santiago, Campus La Florida, Avenida Walker Martínez 1360, Chile. [b] Facultad de Química y Biología, Universidad de Santiago de Chile, Av. Bernardo O'Higgins 3363, Santiago, Chile. *Corresponding author: aaracena@udla.cl (A. A.) and moises.dominguez@usach.cl (M. D.)

Electronic Supplementary Material

Contents

1. Calculation protocol of %CT with the AOMIX software……………………………………….p2
2. Fragment percentage of electron densities in the HOMO and LUMO…………………..p4
3. Quinoidal mesomeric representations…………………………………………………………….p5
4. Natural Transition Orbitals for S0 S1 for dyes 1, 2 and 10………………………………….p7
5. Protocol to obtain BLA and BOA indices…………………………………………………………….p8

1. Calculation protocol of %CT with the AOMIX software.

The geometry optimization of the complete molecule X–Y was carried out with G09. First, the optimized structure must be calculated by a single point calculation using the command line: #P method/basis set test Pop=(Full,NPA) SCF=Tight IOp(3/33=1) nosymm. Then the complete molecule is separated into two fragments X and Y (Fig. S1-S2), and a single-point calculation using the same command line is made for each fragment X and Y. This process yields three output files: molecule.log, fragmX.log, and fragmY.log. All these files are placed into the AOMIX folder. Starting AOMIX software will take as input to complete molecule.log name followed by FO keyword to perform AOMIX-FO calculation. When the calculation is finished, the gross contributions (%) for HOMO and LUMO are printed in the composition of the molecular orbital section of the AOMix-MPA-fr2.txt file.

This protocol was applied to all dyes **1**–**13** in the four media: gas phase, chloroform, dimethyl sulfoxide, and water solution. The net results are shown in Table S1.

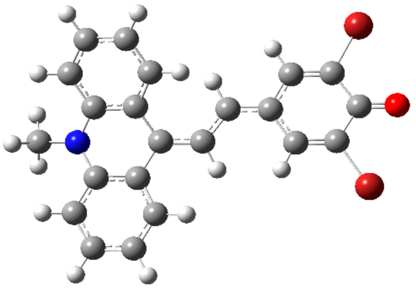

X

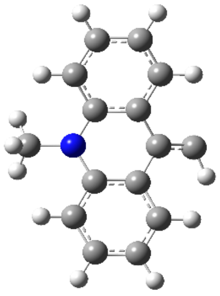

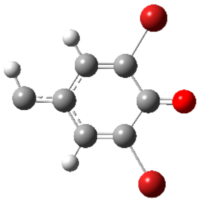

Y

Figure S1 Example of pyridinium phenolate stilbene type (complete molecule, top) and its respective fragmentation (fragmX (**X**), and fragmY (**Y**)).

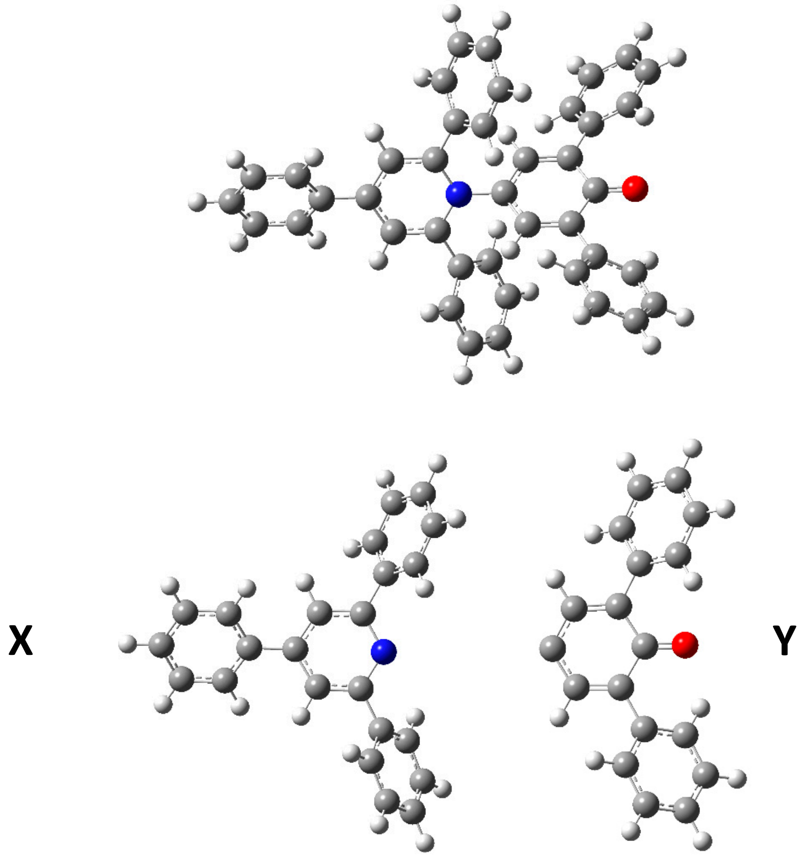

Figure S2 Example of pyridinium phenolate *p*-betain type (complete molecule, top) and its respective fragmentation (fragmX (**X**), and fragmY (**Y**)).

2. Fragment percentage of electron densities in the HOMO and LUMO

Table S1. Fragment percentage of electron densities in the HOMO and LUMO of dyes **1**–**13**.

Dye	X–Y	Gas-phase		CH_3Cl		DMSO		H_2O	
		%H	%L	%H	%L	%H	%L	%H	%L
**1**	X	45.8	44.8	42.3	47.8	40.0	49.8	40.3	49.6
	Y	54.2	55.2	57.7	52.2	60.0	50.2	59.7	50.4
**2**	X	50.5	32.0	46.7	36.2	44.8	38.5	44.7	38.7
	Y	49.5	68.0	53.3	63.8	55.2	61.5	55.3	61.3
**3**	X	58.5	20.8	55.3	22.3	53.7	23.1	53.6	23.2
	Y	41.5	79.2	44.7	77.7	46.3	76.9	46.4	76.8
**4**	X	40.7	45.6	46.7	49.9	34.7	52.1	34.6	52.3
	Y	59.3	54.4	63.3	50.1	65.3	47.9	65.4	47.7
**5**	X	44.5	50.8	40.1	55.6	37.6	58.6	37.5	58.8
	Y	55.5	49.2	59.9	44.4	62.4	41.4	62.5	41.2
**6**	X	50.6	46.1	46.5	50.0	44.6	52.1	43.4	53.3
	Y	49.4	53.9	53.5	50.0	55.4	47.9	56.6	46.7
**7**	X	28.6	67.2	26.0	70.2	24.6	71.9	24.5	72.0
	Y	71.4	32.8	74.0	29.8	75.4	28.1	75.5	28.0

**8**	X	27.5	69.2	24.5	72.7	22.5	75.0	22.4	75.2
	Y	72.5	30.8	75.5	27.3	77.5	25.0	77.6	24.8
**9**	X	36.1	63.7	33.9	65.8	32.7	66.9	32.6	67.0
	Y	63.9	36.3	66.1	34.2	67.3	33.1	67.4	33.0
**10**	X	17.4	83.4	14.6	86.1	13.1	87.6	13.1	87.7
	Y	82.6	16.6	85.4	13.9	86.9	12.4	86.9	12.3
**11**	X	30.5	81.9	27.2	84.2	25.5	85.2	25.4	85.2
	Y	69.5	18.1	72.8	15.8	74.5	14.8	74.6	14.8
**12**	X	34.7	68.1	32.5	69.6	31.5	70.2	31.5	70.3
	Y	65.3	31.9	67.5	30.4	68.5	29.8	68.5	29.7
**13**	X	40.8	62.4	38.0	63.9	36.6	64.8	36.5	64.8
	Y	59.2	37.6	62.0	36.1	63.4	35.2	63.5	35.2

3. Quinoidal mesomeric representations

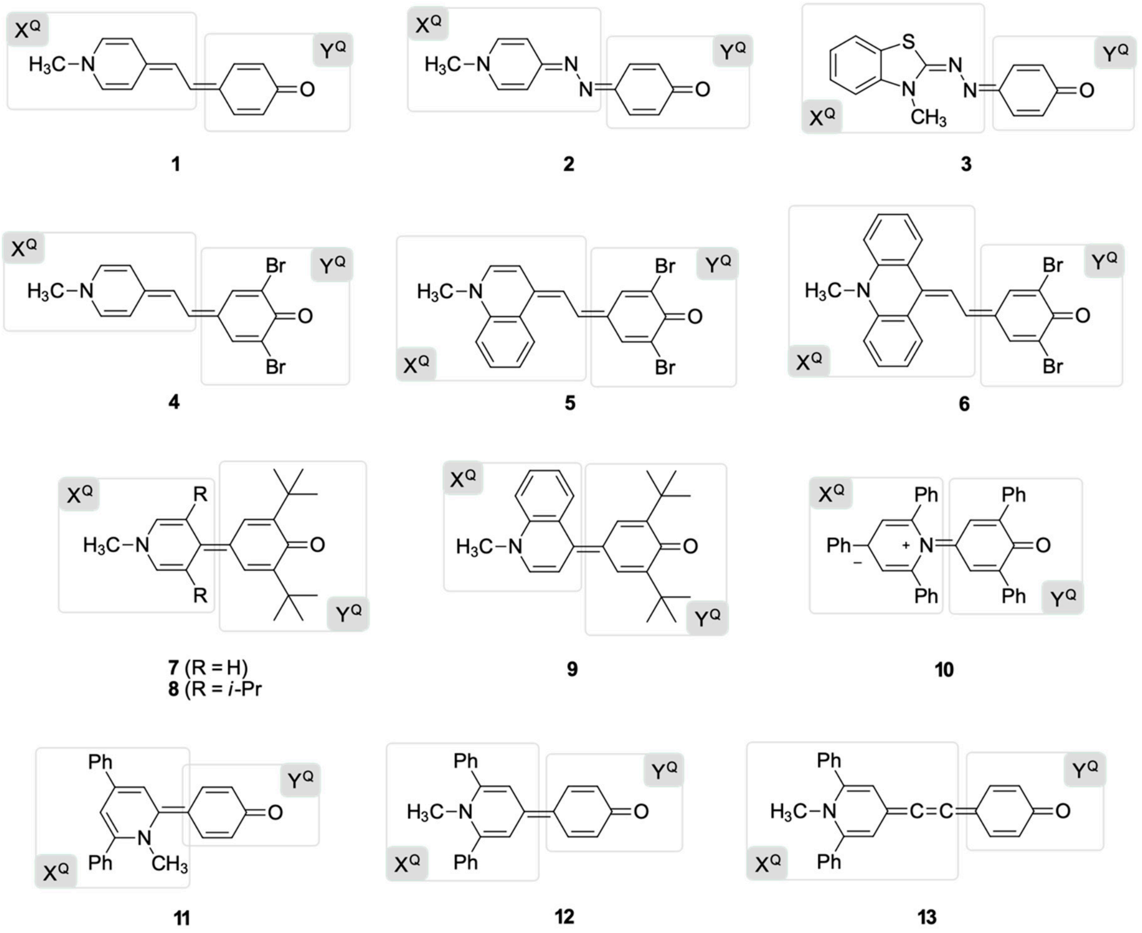

Figure S3 Molecular structures of the solvatochromic phenolate dyes X–Y studied in this work. All molecules are represented with their canonical quinoidal formulae (Q), indicating how they were split into the X^Q and Y^Q.

4. Natural Transition Orbitals for $S_0 \rightarrow S_1$ for dyes 1, 2 and 10.

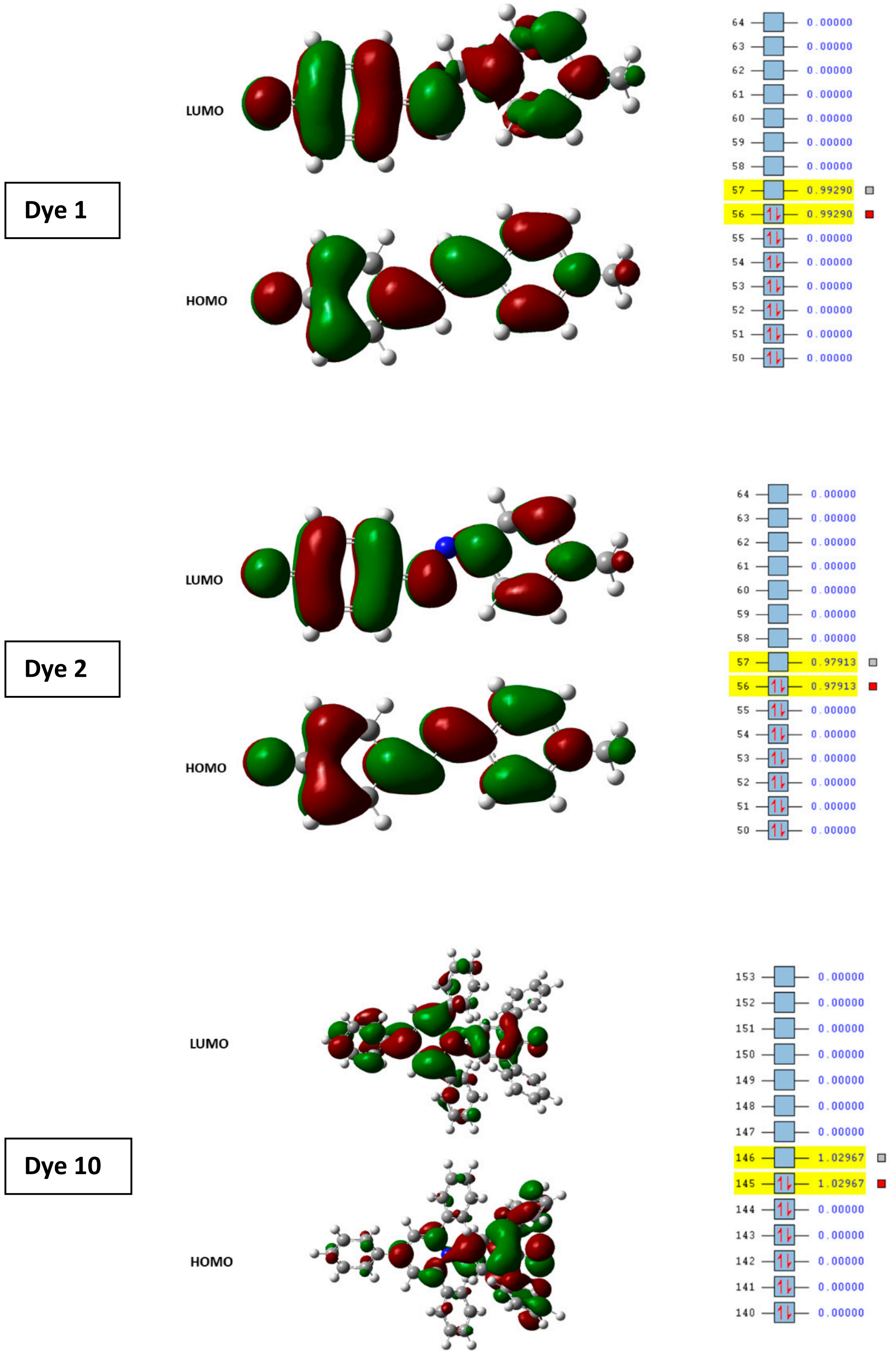

Figure S4 Natural Transition Orbitals (NTOs) and their occupancies involved in the transition $S_0 \rightarrow S_1$ for dyes 1,2 and 10.

5. Protocol to obtain BLA and BOA indices.

The .fchk file for each molecule in the gas phase and in the continuum was analyzed for Multiwfn version 3.8 software in the option 200 and then in option 18. First it is necessary select the atoms of the path of our conjugated system using Gaussview 5.0.8 in the option select atom by rubberband. Then in the menu edit - atom select we will find the chosen path by us. When the option 18 in Multiwfn 3.8 (Calculate bond length/order alternation (BLA/BOA)) is chosen you must add the indices of the atoms in the path obtained from Gaussview and after the Index of the atom at the beginning side and ending side of the chosen path. This last step will allow the appearance of the BLA an BOA indices from the chosen path.
